# Perceptions of Service Climate in a Canadian healthcare system

**DOI:** 10.1177/08404704231171006

**Published:** 2023-05-10

**Authors:** Claudia Steinke, Helen Kelley, Olu Awosoga, Ruth Ann Rebutoc

**Affiliations:** 1 120459 University of Lethbridge, Lethbridge, Alberta, Canada.; 2 70401 University of Calgary, Calgary, Alberta, Canada.

## Abstract

Perceptions of Service Climate in healthcare organizations are important because of their linkages to patient and organizational outcomes. This article presents findings from survey data collected from frontline nurses (n = 275) in Canada who were working in a provincial healthcare system that had recently undergone significant structural changes. The findings indicate that frontline nurses held a neutral view of the Service Climate overall but a strong and negative perception of Managerial Service Practices. The results suggest that some service practices existed in nurses’ working environments; however, improvements could be made in the areas of recognizing and rewarding those who consistently provide high levels of quality service. This has implications for not only continuous quality improvement but also for the patient and staff experience in healthcare.

## Introduction

In this article, we draw on the theory of Service Climate^1^ to make genuine and sustainable improvements to the quality of service delivered in healthcare. Service Climate is “the degree to which management emphasizes service quality in all its activities”^2^ (e.g. policies, practices, and procedures) including employee behaviours that are expected, supported, and rewarded^2^ in providing quality service. Before we delve into the concept of Service Climate, it is important to reflect on our understanding of “service” in healthcare.

In public sector healthcare, the definition of “service,” as per the service management literature, is an ambiguous term—as a great majority of healthcare providers relate more to the concept of providing “care” than “service.” The healthcare system is unique in the product it provides—personal health services. Although healthcare is described as the “world’s largest service”^3-7^ and is arguably the most personal service people consume, there is wide variation in the quality of service provided. This variation is largely because in healthcare, people interact with the system at different times, for different reasons, in different settings, with different levels of urgency, and are met with different service providers with different priorities. The less the consistency placed on the value of “service,” the more diverse people's experiences will be.

In healthcare, the patient’s main concern is for a positive clinical outcome; however, the processes by which that outcome is achieved are in some ways neglected to the detriment of the patient and the organization. The characteristics that shape the experience of “service,” beyond technical competence, are rarely discussed in medical or health management literature.^7^

Service refers to the myriad characteristics that shape the experience of healthcare for patients and their families other than the technical quality of diagnostic and therapeutic procedures. Correct medications, suture placements, and the efficient reduction of a shoulder dislocation are issues of technical quality. Promptly responding to the patient’s needs and answering questions to the patient’s satisfaction in a clear, friendly, culturally relevant, and easily understood manner are components of service quality. In the hospitality sector, service quality is commonly measured on aspects of tangibles (i.e. professional dress, quality of the physical environment), and the reliability, assurance, empathy, and responsiveness of the staff. We rarely hear of such aspects of service measured in public sector healthcare in Canada.

**Figure 1.**
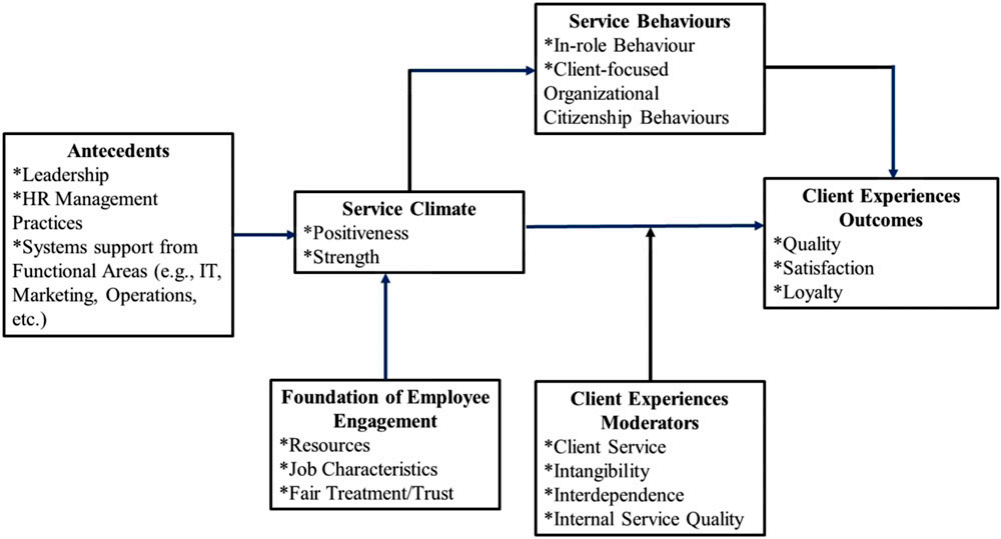
Bowen and Schneider’s Service Climate framework.^8(p 6)^

The investigation of Service Climate, based on Schneider’s^1-2,8^ early conceptualization, has generated a prolific body of research largely in the service management and service marketing fields, less common in healthcare. This work has developed Service Climate measurements and their related attributes (e.g. positiveness, strength). The concept of Service Climate is distinctly different from other constructs such as job satisfaction, organizational culture, and organizational climate.^2^ As described by Bowen and Schneider,^8^ “service climate is contextually service specific, descriptive, and a ‘collective’ service emphasis of the context.” Bowen and Schneider^8^ developed a variance framework that depicts the validated theoretical associations published in the Service Climate literature. This framework describes the antecedents that promote a positive Service Climate for employees, the mediators, and moderators of the associations between Service Climate and people’s experiences, and some outcomes for clients based on their experience of services (see Figure 1). In essence, developing a strong and positive Service Climate within an organization result in more positive service experiences for people, which in turn leads to more positive organizational outcomes (e.g. financial benefits).

In some of the studies, nurses’ perception of Service Climate was positively associated with their units’ efforts expended on tasks and contextual performance^9^; patient satisfaction^9^; patient-centred care^10^; customer-oriented behaviour^11,12^; customer service orientation (referred to as customer-oriented surface trait)^12^; and organizational commitment.^12^ In other studies, Service Climate fully mediated the relationship between service quality and client satisfaction.^13^ Based on data collected from nurses and their immediate supervisors (i.e. charge nurses), Walumbwa et al.^14^ found that structured leader behaviour produced a high-quality Service Climate, which in turn affected collective work-unit meaningfulness. Additionally, both managerial practices and physical design were found to predict a strong Service Climate.^13^ Service Climate in the above studies was measured using a molar aspect (also referred to as global aspect) measure. Typically, the research adopted or adapted Schneider et al.’s^15,16^ Global Service Climate scale, which includes a set of summary items of Service Climate. What has not been investigated in a healthcare environment is the examination of a composite scale of Service Climate composed of diverse service practices, or facets, of the work environment.

## Purpose

In this article, we report the findings from a study that assessed frontline nurses’ responses to questions about Service Climate practices within their work environment. We were interested in understanding what service practices may or may not improve the quality of service delivered on the frontlines of healthcare within a system that is constantly undergoing organizational change.

## Method

The survey was reviewed and approved by a provincial regulatory college. Once approved, the contact information was released for those individuals (potential participants) that met the following criteria: (i) registered as a nurse within the province; (ii) consented to participate in research; (iii) held the position of staff (frontline) nurse; and (iv) worked in the hospital setting (rural or urban). The information technology team of the regulatory college extracted the names and e-mail addresses of the subset, and the researchers began solicitation of potential participants through e-mail by sending a brief statement about the intended purpose of the study followed by a letter of informed consent and a link to the survey. The survey was hosted on-line through the platform SurveyMonkey and took about 10 minutes to complete. The survey contained a series of previously validated measurement items,^15,16^ based on a five-point Likert scale, adapted from the extant literature and nine demographic items. The measurement items were adapted to fit the healthcare context (e.g. the word patient replaced the word customer). Following the ethics protocol approved by the University of Lethbridge Human Subject Research Committee, survey participants were informed that a submitted survey was viewed as their consent to participate. The data collected were aggregated for analysis and summarized, so no personally identifiable information would be published. A total of 275 completed surveys were received from frontline nurses, and this is from a total of 555 respondents. The other 280 respondents were registered nurses that did not work on the frontlines in healthcare but rather held the position titles of either Director/Assistant Director, Consultant, Clinical Nurse Specialist, Manager/Assistant Manager, Chief Nursing Officer/Chief Executive Officer, and Other. The findings presented in this paper focus solely on the perspectives of frontline nurses. The sample characteristics are outlined in Table 1.

**Table 1.** Sample characteristics.

Characteristics	Categories	N^a^	%	Mean	Standard deviation
Gender	Female	262	95.3		
	Male	11	4.0		
Education	College/Technical	64	23.3		
	Undergraduate	96	34.9		
	Graduate	94	34.2		
	Other	19	3.3		
Yrs. in current position				10.3	9.6
Yrs. in healthcare				17.4	11.2
Geographical area^b^	Urban	223	81.1		
	Rural	44	16.0		

^a^Percentages less than 100.0% are due to missing data.

^b^The sample proportion of urban vs. rural geographic area is representative of the population.

The survey items were adapted from previously validated scales reported in the literature. Schneider and colleagues^15,16^ identified three subscales, based on organizational service-focused practices that composed the composite scale of Service Climate: Customer Orientation, Customer Feedback, and Managerial Practices. A two-step process was used to create the composite scale for Service Climate: (1) composite scales for each of the subscales were created by averaging the scores of their associated items and (2) these composite scales were then averaged to create the overall scale for Service Climate.

The “positiveness” attribute of Service Climate and its subscales is a “high mean value” or the “mean of the Service Climate survey items on employee perceptions of service-focused practices and rewards.”^8^ A higher mean value indicates a positive perception of the Service Climate practice. The “strength” of Service Climate is “one in which employees have consensus on what the climate is.”^8^ A low variance in the employee’s perception of the Service Climate practice indicates a strong, or high strength, climate.^8^ The relevance of both “positiveness” and “strength” of Service Climate is related to the organization’s client experience. Specifically, a high mean level (or “positiveness”) of Service Climate has a significant, positive association with client experiences. A low variance (or high “strength”) value has a significantly higher association with client experiences.^8^

## Findings

A confirmatory factor analysis was conducted to confirm that the subscale items, or the factor structure, provided evidence of construct validation for each of the three subscales of Service Climate. The confirmatory factor analysis demonstrated that the subscale items loaded on their associated subscales; however, two items were dropped due to cross-loading. Cross-loading occurs when an observed variable has a high loading on more than one factor, indicating that it is influenced by multiple latent or dormant variables. The summated scales demonstrated acceptable levels of internal consistency (Cronbach’s alpha values greater than .70)^17^ see Table 2. Next, a reliability analysis was performed to determine the measurement consistency of each subscale. The results from the reliability analysis of the summated scales demonstrated acceptable levels of internal consistency (Cronbach’s alpha values greater than .70)^18^

**Table 2.** Reliability and descriptive statistics.

Theoretical concepts	Construct scales^a,b^	# items	Cronbach’s alpha α	Mean	Std. dev.	Variance
Service Climate	Service Climate (composite measure)	3 subscales	.812	3.02	.72	.52
	-Service Feedback	4	.833	3.30	.82	.68
	-Service Orientation	7	.914	3.45	.89	.80
	-Managerial Practices	4	.750	2.21	.82	.67

^a^The mean-item summated scales were based on a Likert scale ranging from Strongly Agree (5) to Neither Agree nor Disagree (3) to Strongly Disagree (1) and Not Applicable (0).

^b^Interpretation of the descriptive statistics was based on a mean value greater than 3.00 as a positive perception; a mean value of 3.00 as a neutral perception; and a mean value less than 3.00 as a negative perception.

Overall, frontline nurses held a strong, neutral (m = 3.02; variance = .52) view of Service Climate and a strong, positive view of two of its practice subscales—Service Feedback and Service Orientation (m = 3.30 and 3.45, respectively), while their view of Service Managerial Practices was strongly negative (m = 2.21; variance = .67) (see Table 2).

The means, variances, and frequencies of the Likert scale of each of the three subscales of Service Climate were examined (see Table 3) to develop a deeper understanding of frontline nurses’ perceptions of various organizational service-focused practices plus observed service behaviours that were expected, supported, and rewarded. Frontline nurses’ perceptions of the four items of the Service Feedback subscale were positive. However, the strength of these items was low, which indicates a lower level of consensus for these practices among frontline nurses. Of particular interest is 47.2% of frontline nurses agreed that patient satisfaction was the number one priority of the organization compared to 21.1% who reported a neutral perception and 31.6% who reported a negative perception (disagree/strongly disagree). This is surprising given that patient satisfaction is one of the key quantifiable performance indicators reported in the healthcare organization under study. A similar pattern was evident for the practice of the supervisor setting high-quality service standards, 44.8% of respondents reported a positive perspective (agree/strongly agree) compared to 30.1% who reported a neutral perspective and 25.0% who reported a negative perspective (disagree/strongly disagree).

**Table 3.** Descriptive statistics for items of Service Climate subscales.

Subscale	Scale item	Mean	Std. dev.	Variance
Service Feedback	Patient satisfaction is the number one priority of our organization.	3.24	1.193	1.424
	My supervisor tells me that high quality service is expected.	3.36	1.068	1.140
	High quality service is emphasized to ensure patient satisfaction.	3.38	1.021	1.043
	My supervisors set definite quality standards for providing good service.	3.19	.992	.984
Service Orientation	My supervisor is committed to improving the quality of service we provide.	3.61	1.103	1.217
	My supervisor appreciates high quality service.	3.88	.993	.985
	I receive adequate support from management to do my job well.	3.10	1.226	1.504
	My supervisor recognizes me for delivering high quality service to others.	3.06	1.182	1.398
	My supervisor shows interest in answering my questions.	3.47	1.120	1.254
	My supervisor supports employees who come up with new ideas on how to improve service.	3.23	1.086	1.179
	My supervisor encourages me to provide high quality service.	3.78	.939	.882
Service Management Practices	If I perform my job well, I receive appropriate recognition and reward.	2.50	1.115	1.243
	If I provide high quality service, I will be rewarded.	2.22	.946	.896
	My supervisor uses rewards to let employees know what kind of job they are doing.	2.11	.887	.786
	Staff satisfaction is the number one priority of our organization.	2.02	.976	.952

Note: Two items were dropped due to cross-loadings.

Our analysis of the practice items for the Service Orientation subscale revealed a different situation. Frontline nurses reported very strong (consensus) and positive (agree/strongly agree) perceptions of their supervisor’s commitment, appreciation, and encouragement of high-quality service. The percentage of nurses who agreed and strongly agreed with those items ranged from 65.6% to 77.2%. Conversely, frontline nurses’ perceptions of receiving the necessary support from management to do their job well (neutral, disagree/strongly disagree = 41.4%), receiving recognition for delivering high-quality service (neutral, disagree/strongly disagree = 55.6%), and receiving supervisory support for suggesting new ways to improve service (neutral, disagree/strongly disagree = 55.4%) were weak (mean > 3.00). These findings suggest that supervisors may incorporate some aspects of what is required for establishing a positive Service Climate, but other aspects were lacking such as providing the necessary support and recognition for delivering high-quality service and supporting ideas to improve service.

Of particular interest were frontline nurses’ strong and negative perceptions of Service Managerial Practices. Over 90% of frontline nurses reported a neutral or disagree/strongly disagree stance about their supervisors providing employees with rewards for doing their job well, and 77% indicated a negative response to receiving appropriate recognition and rewards for doing their job well and providing high-quality service.

## Discussion

Understanding the diverse service practices that make up a strong Service Climate is imperative to genuinely improve the quality of services delivered on the frontlines of healthcare. As noted by Hong et al., “… Service Climate is a more proximal reflection of an organization’s philosophy and practices than other outcomes.”^18^ Assessing nurses’ perceptions of Service Climate can provide a diagnosis of the effectiveness of the organization’s strategy for developing a culture of service quality.^8^

The findings of this study provide evidence that frontline nurses have a strong and neutral perception of Service Climate based on subscales that speak to the organizational facets of Service Feedback, Service Orientation, and Service Management Practices. Frontline nurses’ overall views of Service Feedback and Service Orientation were strong and positive, whereas their views of Managerial Practices were strong and negative. Of interest, frontline nurses’ perceptions of these diverse service practices were mixed and their strength (i.e. consensus) and positiveness varied.

In terms of Service Feedback, patient satisfaction was felt to be a priority in the organization but not the number one priority. Providing high-quality service to patients is somewhat expected but not emphasized as much as it could or should be. If a healthcare organization truly wants to be known for providing high-quality service, Service Feedback practices must be emphasized, expected, and reinforced daily, and definite quality standards must be set. A practical strategy to improve in this area is to have someone such as the Unit Manager speak with the patients in their department every few days to assess how their experience has been thus far. This will provide valuable feedback and identify areas needing improvement along with areas of strength. This will also show staff that the quality of the service experience matters. Another strategy involves setting up a Service Experience Team. This could be a team of five to seven people that are familiar with the department and its operations. Stakeholders could include the following: a physician, a registered nurse, a licensed practical nurse, a healthcare aide, a past patient, a family member of a past patient, a member of Environmental Services, a unit clerk, a volunteer, and the manager specific to the department, who could gather feedback on the quality of the service provided, suggest and implement ideas for improvement. In addition, during the discharge process, the nurse reviewing the discharge plan with the patient could inquire about the patient’s experience of service and relay that information back to the Service Experience Team.

In terms of their work environments having strong a Service Orientation, nurses agreed their immediate supervisors were committed to improving the quality of service, and it was encouraged and appreciated. However, nurses felt they were not being recognized when they provided high-quality service, nor were they provided with the necessary support to do so. This could be interpreted as "yes, we want you to provide a level of high-quality service to patients and yes, we appreciate you doing this, but we cannot provide you with the necessary support to do so." These findings suggest that more attention should be placed, at a much deeper level, on what it means to provide high-quality service and a clear vision developed as to what this looks like. It must become ingrained in the culture of the organization. Striving to provide the additional support(s) needed to make this happen (e.g. more staff, more time, more tools) on a consistent basis will better enable nurses to do this. Taking the time to recognize those that do provide high-quality service and those that go above and beyond to make the experience of healthcare as positive as possible (e.g. professional, pleasant, comforting, efficient, and effective) will help to instill a strong Service Orientation.

Frontline nurses’ perceptions of Managerial Practices were strongly negative. The nurses felt that organizational service-focused practices of recognizing and rewarding jobs well done and providing high-quality services to patients did not exist. These frontline professionals did not perceive staff satisfaction as a priority of their organization. These negative perceptions suggest that the organization and its managerial teams should evaluate current human resource practices to look for ways to improve recognition and rewards for providing frontline high-quality service. This finding highlights the need for managers to find a much better way to recognize nurses for a job well done, let them know, and show them how much they’re appreciated. When you see a nurse that goes above and beyond in providing high-quality service to patients, be sure to tell them. The more specific the feedback, the better. This will also help to set definite quality standards for providing high-quality service in healthcare.

According to the Service Climate framework and validated findings, managers of healthcare organizations who wish to increase the level of service quality in their departments should focus on developing a climate for service. This can be done by reflecting on one’s own service-oriented mindset and actions, along with the service-oriented policies, practices and procedures of the organization, and the service behaviours of employees. As reported by Hong et al.,^18^ service-oriented human resource practices, service-oriented leadership, and transformative leadership can directly influence employees’ perceptions of Service Climate. Our study found that certain service-focused practices appear to not have existed, which led to a poor/weak Service Climate, and, in turn, may have negatively influenced the patient service experience. According to Schneider et al.,^16^ when Service Climate is weak, regardless of whether it is positive or negative, the prediction of service behaviour is less reliable. An organization with a strong Service Climate (i.e. a place where events are perceived the same way and expectations are clear) should produce uniform, consistent behaviour from the people in that service setting.^19^ In other words, in weak climate conditions, regardless of the degree of the climate perceptions, predictions of behaviour will be less reliable than when the climate is strong. When trying to improve the level of service quality in an organization or department, creating the conditions that will facilitate a strong climate for service is paramount.

## Limitations of the research

This study has some limitations that must be noted. Collecting data via a cross-sectional survey (i.e. a representative subset of the sample population during one moment in time) limits the ability to draw causal conclusions. For interest’s sake, the researchers initially tried to solicit participants from the provincial health system but ended up contacting the provincial regulatory college for assistance. The service experience with the latter was efficient and effective. A second limitation relates to the external validity of the results to other areas of public sector healthcare (e.g. public health and allied health). Research conducted in other public sector healthcare areas would enhance our findings' generalizability. Thirdly, the impact of the unionized environment in healthcare needs to be considered—the question stands as to whether a highly unionized environment impacts the ability to develop and sustain a strong Service Climate.

## Future research

Conducting this research in other areas of public sector healthcare such as the community or continuing care setting would be valuable, as would conducting a comparison study between similar environments within public and private care healthcare. Assessing the impact of a highly unionized environment on the provision of recognition and rewards is a question that has been posed to the authors. An additional step in this research is to assess perceptions of service quality by comparing the quality of service delivered (as perceived by nurses) with the quality of service received (as perceived by patients).

## Conclusion

In closing, this study addresses a gap in the literature by exploring associations of Service Climate within a public sector healthcare system. Several practical suggestions, based on the theories of Service Climate, are presented to help shape how organizations may establish and strengthen a climate for service in healthcare.
